# Multilayer framework for digital multicomponent platform design for colorectal survivors and carers: a qualitative study

**DOI:** 10.3389/fpubh.2023.1272344

**Published:** 2023-12-05

**Authors:** Samar J. Melhem, Reem Kayyali

**Affiliations:** Department of Pharmacy, School of Life Sciences, Pharmacy and Chemistry, Kingston University London, Kingston upon Thames, Surrey, United Kingdom

**Keywords:** colorectal cancer, e-health, digital health, supportive care, survivors, caregivers, digital multicomponent platform, user-centered design

## Abstract

**Background:**

The advent of eHealth services offers the potential to support colorectal cancer (CRC) survivors and their informal caregivers (ICs), yet research into user needs and design requirements remains scant. This exploratory qualitative study addresses this knowledge gap by focusing on the development of a Digital Multicomponent Platform (DMP) designed to provide comprehensive support to these populations.

**Aims:**

The objective of this research is to use qualitative methodologies to identify key user needs and design requirements for eHealth services. It seeks to propose and apply a multi-tiered framework for creating a DMP that encapsulates the needs of CRC survivors and their ICs.

**Methods:**

Skype-based focus groups (FGs) were utilized to gather qualitative data from CRC survivors and ICs. This approach served to elicit crucial themes integral to the design of the DMP. A multi-tiered framework was subsequently developed to integrate user-centered design (UCD) principles and requirements with predetermined outcomes, eHealth services, and IT infrastructure.

**Results:**

The first stage of the analysis identified five crucial themes: (1) the importance of healthcare system interaction via eHealth, (2) interaction between healthcare providers and peers, (3) lifestyle and wellness considerations, (4) platform content and user interface requirements, (5) caregiver support. The second stage analysis applied the multi-tiered framework, to determine the DMP that was conceptualized from these themes, underscores the significance of personalized content, caregiver involvement, and integration with electronic health records (EHRs).

**Conclusion:**

The study offers novel insights into the design and development of digital supportive care interventions for CRC survivors and their caregivers. The results highlight the utility of user-centered design principles, the significance of personalized content and caregiver involvement, and the need for a unified health data platform that promotes communication among patients, healthcare providers, and peers. This multi-tiered framework could serve as a prototype for future eHealth service designs.

## 1 Introduction

Colorectal cancer (CRC) ranks among the most prevalent cancers worldwide (1). Recent advancements in therapeutic interventions have amplified survival rates among patients diagnosed with this cancer (2). These CRC survivors often encounter a multitude of challenges related to their mental wellbeing, fiscal state, and health-related quality of life (HRQOL) resulting from the cancer and its treatment. Challenges such as anxieties about recurrence, surgery side effects, or ostomy management, specific to their treatment, have also been documented (3–6). Consequently, contemporary cancer care has pivoted toward a comprehensive approach, encompassing multidisciplinary care coordination, surveillance, symptom and psychological support, health maintenance, and survivorship care (7–9). Survivorship care has been incorporated into the American College of Surgeons Commission’s accreditation standards, and guidelines advocate for an expansive survivorship treatment beyond mere cancer surveillance (10–12).

Cancer patients’ perceived requirements for supportive care can be categorized into five principal segments: psychological needs, health system and information needs, physical and daily living needs, patient care and support needs, and sexuality needs (13). The efficient provision of survivorship care plans (SCPs) and other supportive care services must negotiate multiple challenges. These challenges stem from a myriad of sources such as busy schedules of doctors hindering accessibility, communication gaps between specialists, lack of synchronization between providers’ workflow and survivorship planning efforts leading to inadequate SCPs. Additionally, evolving supportive care needs throughout the treatment and scarcity of resources for healthcare practitioners to design a personalized, patient-centered approach add to these challenges. Patients often delay seeking medical help due to unawareness about SCPs and supportive care services, and the lack of knowledge and training might limit healthcare providers’ ability to develop and implement SCPs. Furthermore, scarcity of medical resources, financial hurdles, and healthcare personnel often limit many patients from accessing effective and optimal support care services.

With increasing integration of healthcare systems and patient engagement, eHealth has emerged as a notable entity. eHealth optimizes communication and collaboration between patients and their care teams to enhance health outcomes (14). The delivery of health services via telehealth or telemedicine incorporates real-time, two-way interaction facilitated by electronic means. The rise in remote clinical services like patient follow-up or management of chronic diseases makes telemedicine a feasible option. “Teleconsultation” or e-consultations employ telehealth technologies to facilitate consultations between patients and primary care physicians (15, 16). In this study, eHealth is used to refer to telehealth/telemedicine, e-consultations, and digital interventions. The multifaceted domain of eHealth spans from decision support to patient education to prognosis and treatment planning, often incorporating Artificial intelligence (AI). The most prevalent form of eHealth in oncology is the Patient Decision Aid (PDA). PDAs, available in digital as well as paper-based forms, aim to equip patients with necessary knowledge for making informed treatment decisions and assessing the value of available options (17). They have been proven to mitigate disagreements during crucial decision-making processes (18, 19). eHealth interventions can enhance patient experience and quality of life across many supportive care domains (20).

E-health is a promising field since it may promote and facilitate health behavior modification as well as aid in illness prevention and management (21). Once developed, digital interventions may be readily sustained due to the minimal costs of ongoing maintenance and deployment. One study showed that new multimedia developments may be leveraged to improve the dissemination of patient education (22). Studies have revealed that web-based health education resources are more effective and patient-satisfying than those delivered in a conventional manner (23). Written material may not be useful for oral communicators or low-literate people. Expert advice for HL interventions includes avoiding a “*one-size-fits-all*” approach (24) and developing materials that improve participant engagement and retention through interactivity, intriguing multimedia features, and ensuring learning using an interactive teach-evaluate-reteach-when-needed algorithm (25).

Despite the potential of eHealth interventions to elevate personalized supportive care, research investigating their efficacy in enhancing patient outcomes compared to traditional supportive care interventions remains limited (26). Limiting supportive care interventions to clinical settings poses a barrier for many patients and raises concerns regarding the feasibility and effectiveness of these interventions in providing supportive care to patients, caregivers, and family members. As challenges often emerge at home and outside working hours, supportive care needs cannot always be met in a physical environment. Therefore, providing patients with supportive eHealth interventions accessible remotely over a secure connection might reduce daily life disruptions, hospital admissions, and healthcare costs (27, 28). Previous research suggests that accommodating survivors’ care preferences enhances their self-perception of autonomy and confidence in managing their health (26, 29), potentially expediting symptom management assistance for patients (30, 31).

Several studies indicate that eHealth interventions for supportive care are safe, acceptable, and effective in improving quality-of-life outcomes for patients (27, 29–34). However, these interventions should be customized in alignment with patient demographics and diagnostic characteristics to cater to the needs of the target population (27, 31, 35). Supportive care interventions fostering trust and reassurance between cancer survivors and their practitioners enhance outcomes for both parties (27, 35). The benefits of supportive care eHealth interventions can vary considerably depending on the needs of cancer patients in specific demographics or at different stages of the disease trajectory (26, 35, 36).

End-user perspectives, i.e., patients and caregivers, need to be incorporated when designing eHealth solutions for supportive care (37–39). E-Health interventions that consider patients’ preferences and expectations for care, along with their health literacy (HL), can enhance patient-centered care by addressing care inequalities, extending access to health information, services, and support, and reducing care disparities (40–42). For eHealth interventions to succeed, it is crucial for patients to take an active role in their care (43). eHealth solutions should be designed considering the complex HL dimensions, healthcare system contextual elements, and by producing responsive and adaptable content suitable to diverse learning styles. Melhem et al. (44, 45), in their recent qualitative and quantitative research on the digital experiences, digital health literacy (DHL) and trends of cancer survivors and the adoption of eHealth apps, highlighted this necessity (44, 45). Patients with low HL may experience information overload while using eHealth applications relying on vast data volumes. On the other hand, the use of eHealth applications in clinical practice has been shown to enhance HL levels in various patient populations, including patients with CRC (46). These findings imply a necessary balance between patient empowerment and information overload for eHealth app developers. For instance, a study employed a PDA to assist CRC cancer patients with low HL in determining their values, significantly improving personal values and uncertainties (47). Additionally, it is important to note that eHealth introduces an additional literacy dimension, namely digital/eHealth literacy, which could further exclude individuals with poor digital literacy as discussed in the subsequent technique (48).

Moreover, involving patients and other end-users in the design process enhances the likelihood of meeting the needs of the intended population and ensures that patients and their families can access the intervention in a setting that suits them best (38). However, patient input is often overlooked during the development of interventions (26, 27, 33, 35). Ventura et al. (32) observed in their systematic review that eHealth interventions are seldom designed using theoretical frameworks, with only 5 of 16 interventions guided by an analysis of patient needs. Similarly, Capurro et al. (49) found that only 11 studies utilized palliative care needs for the design of an eHealth intervention. Theory-informed intervention designs can be used to elucidate outcomes. Comprehensive eHealth models of care and guidelines are necessary to ensure successful supportive care interventions (34). The burgeoning reliance on digital technologies in cancer treatment gives rise to legitimate concerns that the digital literacy gap might aggravate existing inequities in cancer care. It is imperative to address this issue as a priority, adopting a multidisciplinary approach to improve digital literacy and developing culturally and linguistically appropriate platforms (49, 50).

This study seeks to leverage previous findings to discern patients’ and informal caregivers’ (ICs) needs for a supportive digital cancer care platform, informed by their experiences.

The objectives of the study are:

1. Identifying user needs and design requirements for eHealth services to assist CRC patients and their carers across the care continuum following primary surgical treatment.
2. Proposing a multi-tiered framework that aligns user-centered requirements and functions with intended outcomes, eHealth services, and IT infrastructure. This framework aims to identify design features and systematize the design process for a Digital Multicomponent Platform (DMP).

## 2 Methods

### 2.1 Ethical considerations

The study was approved by Kingston University’s ethical principles for scientific research (approval number/1416) and Jordan University Hospital’s (JUH) Internal Review Board (IRB), protocol ID (10/2019/8990).

### 2.2 Design and setting

This exploratory study, based on a qualitative and descriptive phenomenological paradigm which prioritizes the nuances of lived experiences and individual perceptions, enabled a more profound grasp of participants’ interactions with online platforms. This paradigm is devoted to comprehending how individuals extract meaning from their engagement with the world, capturing the emotional and interpretive aspects that shape their subjective realities (51), the study utilized independent online focus groups (FGs) with patients and carers. The study took place from March to June 2020, employing Skype as the medium of interaction. Participants were recruited from JUH, a major tertiary care Center located in Amman, Jordan.

The FGs were structured to stimulate brainstorming and interchange of ideas, facilitating substantive discussions (52, 53). Given prior research identifying DHL as the sole independent determinant of eHealth app use and receptivity to information among CRC survivors (44), the inclusion of informal carers as digital mediators was justified. Further, it is worth noting that past studies have highlighted age as an independent variable influencing internet information utilization among CRC survivors (54).

### 2.3 Data collection

#### 2.3.1 Participant eligibility and recruitment

##### 2.3.1.1 Colorectal cancer survivors’ FGs (*n* = 3)

The study utilized a convenience sample comprising ambulatory CRC survivor’s post-curative surgical therapy. Participants qualified if they were: (1) Aged 18 or above, (2) Diagnosed with CRC, having completed curative therapy and currently in follow-up or surveillance stage, ideally between 2 to 6 years post-treatment, (3) Deemed clinically stable by their healthcare team, and (4) Proficient in Arabic with the capacity to provide informed consent.

Eighteen potential participants were initially identified by two oncologists and contacted by a medical team member. Subsequently, the primary investigator (SJM) followed up with additional information and clarifications. Interested patients were provided with PIS (provided in Supplementary Table S1). Despite five declining participation due to unfamiliarity with online platforms (Skype), and three more withdrawing during the online discussions, 10 participants successfully enrolled and provided consent via email or WhatsApp. Thematic saturation guided the assessment of the number of interviews required, with FGs concluded when no novel themes surfaced (55). Additionally, saturation was based on the concept that conducting two to three focus groups in a relatively homogeneous population using a semi-structured guide can capture at least 80% of the topic’s themes, including the most frequently discussed ones. It is anticipated that 90% of the themes can be identified within three to six focus groups (14). Demographic and clinical data were collected from patients’ electronic medical records.

FGs, organized into small groups of 3–4, had a median duration of 2.5 h. Conducted via Skype in Jordanian Arabic, discussions were recorded, transcribed verbatim, and subsequently translated into English by the first author (SJM), with transcripts reviewed by the two bilingual authors (SM and RK). Participant anonymity was preserved, with access to data limited to the research team. The focus group topic guide underwent a pilot test with a nurse who is also a cancer survivor to evaluate its flow, format, and question clarity. This pilot data was excluded from the final analysis.

##### 2.3.1.2 Informal carers FGs (*n* = 3)

Similar to the patient groups, online FGs with informal carers were conducted in small groups of 3–4 via Skype. Twenty-one potential participants were initially contacted by a medical team member and informed about the study’s objectives. SJM addressed additional queries from the 10 carers who agreed to participate before providing them with the PIS (Supplementary Table S1) and informed consent form. Eligible carers were adults aged 18 or above, fluent in Arabic, and currently caring for a CRC patient.

#### 2.3.2 Focus groups’ topic guide

The study employed a detailed topic guide, provided in Supplementary Table S2, to extract insights from cancer survivors and caregivers on their use and perception of mobile applications. It consisted of 7 main sections with open-ended questions with prompts. The topic guide was developed using *a priori* framework and a literature review, with a focus on user-centered e-health technologies to support CRC cancer survivors and their ICs (56–58). It began by exploring participants’ existing app usage, followed by assessing the perceived value and benefits of mobile apps in supporting cancer care. The guide solicited participants’ desired features and informational needs for a proposed health app, covering areas such as disease management, appointment tracking, and nutrition. It also addressed the app’s potential in enhancing follow-up care, improving communication with healthcare professionals, and aiding medication management. Additionally, it delved into the role of the app in providing emotional and social support, examining the feasibility of features such as support groups and personal experience sharing platforms. The guide investigated potential drawbacks of a cancer care app and evaluated the proposed features’ value across various disease stages.

The potential of the app to augment or replace traditional consultations was also explored. The guide concluded by inviting any additional feedback, ensuring a comprehensive understanding of user needs for the design of an effective digital solution.

### 2.4 Data analysis and reporting

The analysis was conducted in two sequential stages, designed to systematically explore and integrate both user-centric (bottom-up approach) and system-level requirements (top-down approach). The first stage was focused on ascertaining user requirements from a bottom-up perspective, employing a qualitative focus group methodology. Thematic data aggregation was accomplished via a Framework Method, comprising a nuanced, five-phase analytical sequence: familiarization, theme framework development, indexing, charting, mapping, and interpretation. This approach is corroborated by the analytical procedure delineated by Gale et al. (59).

Transcripts of the focus group discussions were meticulously transcribed, anonymized, and analyzed using NVivo 12 software to facilitate data management tasks, such as indexing and charting. A hybrid deductive-inductive analysis was adopted for transcript analysis. Inductive coding was carried out to derive all first order and empirical coding from the data using open (unrestricted coding) and theme refinement to glean emergent themes from the data. Conversely, deductive coding employed pre-established themes and codes, informed by extant literature (12, 40, 60, 61–67) and post-priori concepts regarding the research subject in the focus group topic guide (Supplementary Table S2).

**Figure 1:**
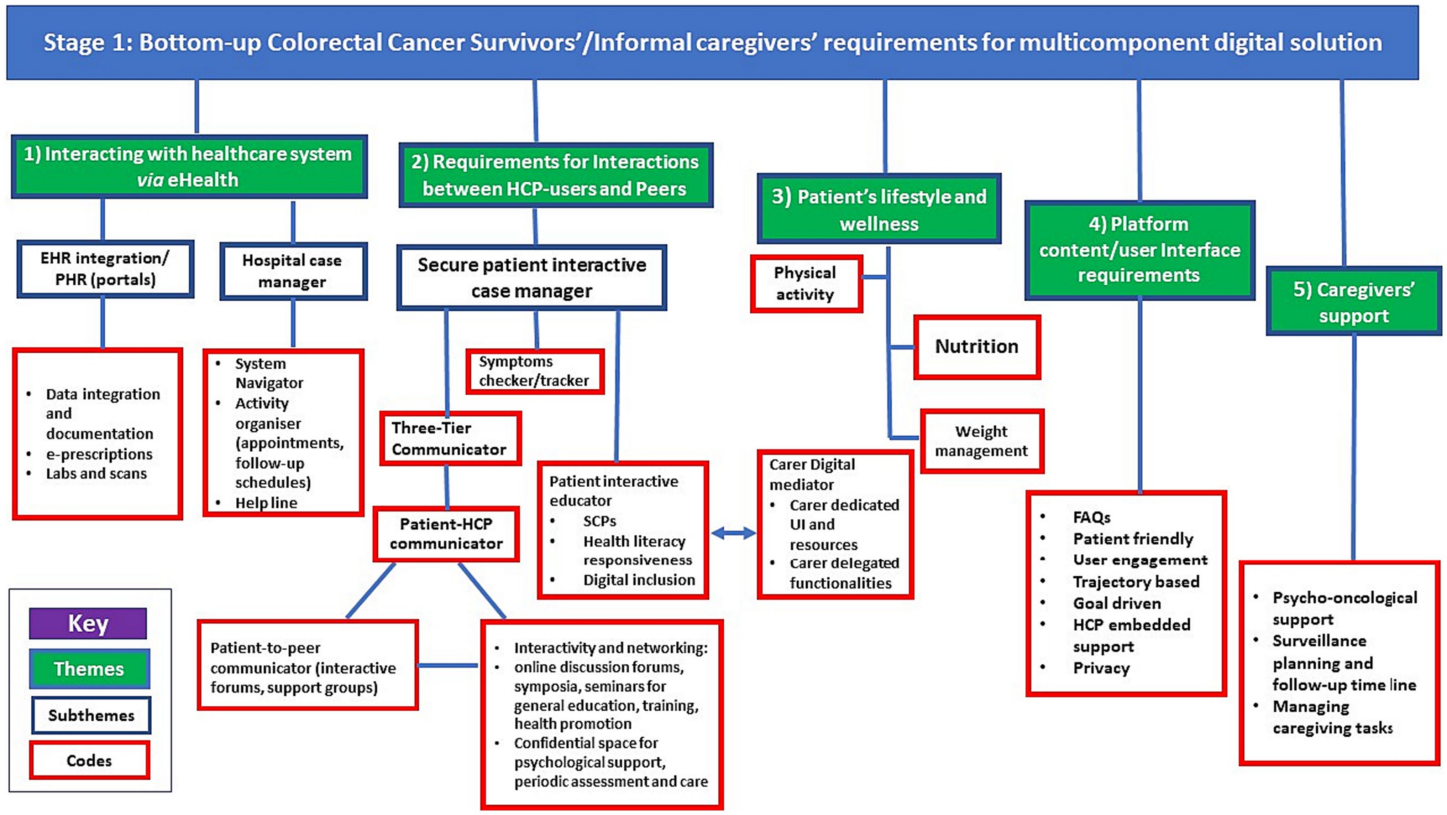
Thematic analysis of bottom-up user requirements of a Digital Multicomponent Platform (DMP). EHR, Electronic Health Records; PHR, Personal Health Record; HCP, Healthcarre professional; FAQs, Frequently Asked Questions.

**Figure 2:**
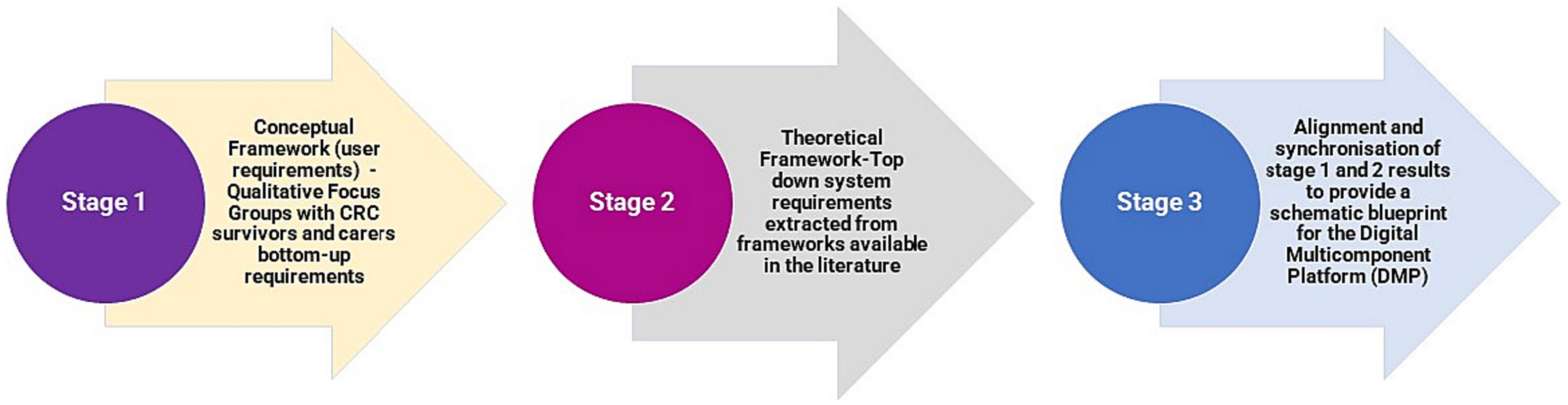
Thematic presentation of the multi-stage methodology adopted.

A preliminary analytical framework emerged from an inductive scrutiny of transcripts, aimed at identifying the specific requirements of CRC survivors and their informal caregivers (ICs). This framework was continually refined through categorization of first-order codes and empirical themes into subthemes and overarching superordinate themes. These were further structured based on post-priori topics identified through a pre-existing body of literature. This stage culminated in a thematic analysis of bottom-up user needs and design requirements, visualized in Figure 1, which served as the foundational layer in a multi-tiered conceptual map (depicted in Figure 2).

The second analytical stage aimed to synchronize these bottom-up user requirements with a top-down multi-layered framework (provided in Supplementary Table S3), as posited by Ayyoubzadeh et al. (58). Their structure comprises four distinct layers: outcome, service, software, and hardware. The outcome layer encapsulates the favorable findings from prior CRC survivor studies, while the service layer offers bespoke solutions and educational resources. The software and hardware layers form the technical infrastructure necessary for service delivery, including various applications, devices, and sensors.

The synthesis of these multilayers–user needs, health outcomes, services, software, and hardware–was organized into a conceptual map for more granular investigation of the domains and features of the DMP in a methodical manner (60). In light of this, Figure 2 unveils a conceptual multi-tiered framework, conceived to harmoniously align user-centric requirements and features with the projected outcomes, e-health services, and information technology infrastructure. This composite framework serves as a schematic blueprint for the collaborative development of a comprehensive DMP, designed to support CRC survivors and their ICs. Supplementary Table S4 illustrates a systematic integration of bottom-up and top-down approaches in the development of an e-health tool for CRC survivors and their carers.

### 2.5 Quality assurance

Quality assurance was maintained by adhering to the systematic audit trail of the framework theme analysis methodology (59). The study’s findings were reported following the COREQ criteria for reporting qualitative findings (Supplementary Table S5) and using a flexible interview schedule (68). Measures such as data recording and transcription, inclusion of direct quotations for referential adequacy, and clear and transparent data gathering, and analysis were followed to ensure integrity, transferability, and credibility (69, 70). The sample and research settings were both described, providing necessary context. Regular meetings with the supervisors allowed for ongoing discussions of the results, contributing to the maintenance of rigor (71). In this paper, quotations are presented in the format of (IC/Survivor number: Focus group number, gender, age in years).

## 3 Results

### 3.1 Participant characteristics

#### 3.1.1 Colorectal cancer survivors’ focus groups

Between April 15 and May 10, 2020, three online FGs were conducted with a total of 10 CRC survivors. The duration of each FG was as follows: FG1 lasted 2 h 54 min, FG2 lasted 2 h 39 min, and FG3 lasted 3 h 10 min.

The participants had an average age of 63.5, with a range of 53 to 74 years. All participants were married, with the majority being female (7 out of 10 participants). Among the participants, 5 out of 10 were classified as stage III survivors. Further details on the characteristics of the participants can be found in Table 1.

**Table 1:** CRC survivors’ characteristic focus groups (*n* = 3).

Variable(s)	CRC survivors (*n* = 10)
	*n* (%)
Age (years)^1^	63.5 (53–74)
Male(s)	3 (30)
Female (s)	7 (70)
Education	
Primary (5–8 years)	0 (0)
Secondary (9–12 years)	1 (10)
High school/collage/diploma (12+ years)	5 (50)
University (14+)	4 (40)
Employment	
Employed	2 (20)
Unemployed (capable/uncapable)	3 (30)
Retired	5 (50)
Cancer type	
Colon	9 (90)
Rectal	1 (10)
Cancer stage	
Stage I	1 (10)
Stage II	4 (40)
Stage III	5 (50)
Stage IV	0 (0)
Treatment modality	
Chemotherapy, surgery	6 (60)
Chemoradiation, surgery	2 (20)
Chemotherapy, surgery, palliative chemotherapy	0 (0)
Surgery	2 (20)
Stoma	
None	2 (20)
Temporary, reversed	5 (50)
Permanent	0 (0)
Unknown	3 (30)
Time since diagnosis (years)	6.5 (2–7)
Comorbidities	
Yes	4 (40)
No	6 (60)

^1^Median (min, max).

#### 3.1.2 Informal carers focus groups

Between 12/6/2020 and 28/6/2020, three FGs with 10 ICs were conducted online. The duration of each FG was as follows: FG1 (2 h 47 min), FG2 (3 h 02 min), and FG3 (2 h 51 min).

Participant characteristics are displayed in Table 2.

**Table 2:** Characteristics of participants of focus groups (*n* = 3).

Variable(s)	Informal carers (*N* = 10)
	*N* (%)
Age (years)^1^	36 (26–62)
Male(s)	4 (40)
Female (s)	6 (60)
Education	
Primary (5–8 years)	0 (0)
Secondary (9–12 years)	0 (0)
High school/collage (12+ years)	1 (10)
University (14 + years)	3 (30)
Masters/PhD (18+ years)	6 (60)
Occupation	
Medical Professional	5 (50)
Engineering, design, tourism	3 (30)
Academia	1 (10)
Housewife	1 (10)
Patients’ cancer type	
Colon	8 (80)
Rectal	2 (20)
Relationship of carer-patient	
Son/daughter	7 (70)
Stepmother	1 (10)
spouse	1 (10)
sibling	1 (10)
Time since patients’ diagnosis until time of study (caregiving experience) (years)	
2–3 years	5 (50)
4–5 years	4 (40)
5+ years	1 (10)

^1^Median (min, max).

### 3.2 Stage 1: thematic analysis of bottom-up user requirements

The bottom-up requirements for a DMP, as identified by CRC survivors and ICs, were classified into five themes: (1) Interacting with the Healthcare System via e-health, (2) Requirements for interactions between HCP-users and peers, (3) Patient’s lifestyle and wellness, (4) Platform Content/User Interface Requirements, and (5) Support for Caregivers. The themes, subthemes, and findings from the framework analysis are depicted in Figure 1.

#### 3.2.1 Theme 1: interacting with healthcare system via e-health

To enhance their access to medical records such as labs, scans, past procedures, and e-prescription refills, CRC survivors and ICs expressed a preference for a digital solution that could overcome the fragmented nature of the healthcare system. They sought a solution that would improve service quality and provide a more connected and integrated user experience. Delivery in the form of digital or electronic services not only improves accessibility, but also empowers users, reduces costs, and streamlines processes. As a result, patients may be able to conveniently share their health information with multiple doctors through a central portal accessible via their mobile device or computer.

*“If the app can track down all the activities that we performed throughout cancer treatments and provide us with records, it would be really valuable, since I often lose some pieces of information that’s why I store all my records in one file and carry it with me when I see any doctor...” (CRC survivor 3, FG3, male, 74 years).*

*“Yes. Helpful if we could add all of the patient’s information, like medical reports and lab tests, and if documents could be downloaded or accessed through an app. “(CRC survivor 1, FG3, male, 67 years).*

In light of the needs of the participants, a digital case manager, such as a hospital case manager that includes a system navigator that uses virtual waiting rooms for more precise timing to meet doctors rather than prolonged wait times was desired.

*“If the app can tell me a precise time like from 2 pm-4 pm waiting to be seen, it would be much more organised for me because I normally go to the hospital in the morning and wait for additional 3–4 h to be seen….” (IC2, FG3, male, 35 years).*

The time spent waiting to be seen by a doctor is often a bottleneck in the healthcare process. It is crucial to efficiently manage the flow of activities and make the most of the available time during hospital visits. Therefore, pre-organizing the patient journey, scheduling and sequencing follow-up arrangements, and coordinating visits can be advantageous, particularly when patients need to move spatially between institutions or see several physicians. By implementing these strategies, HCPs can optimize the use of time and enhance the overall patient experience.

*“Because the steps involved in following up with a hospital visit do not always happen in the same order (e.g., getting lab results, waiting for the doctor, being referred to another clinic, or signing up for tests at a different facility), it’s helpful if patients are informed in advance of what needs to be done before they arrive.” (IC4, FG2, female, 41 years).*

*“The software would be great if it allowed me to organise my visit, arrange my activities, and perform some things remotely, saving me time.” (CRC survivor 1, FG3, male, 67 years).*

*“Since I’ve been having treatment at three different hospitals and the systems are all different, so I get lost and have to waste time waiting, and I do not have to take off a day of work to do it. “(IC2, FG3, male, 35 years).*

*“I have to travel to the hospital each time to obtain the instructions, so if they sent me the prescription and information on how to prepare for the colonoscopy, it would save me a lot of time.” (CRC survivor 1, FG3, male, 67 years).*

Some users have commented that if the app is updated to accommodate patients’ specific clinical needs and provide them with expedited access to care, it has the potential to greatly enhance the quality of their follow-up experience.

*“I’m lucky that the consultant gave me his number so that when I come to hospital, he will let me inside swiftly and I do not have to because he knows my case, but I’m too ashamed to tell the nurses or anyone that I had rectal cancer surgery because I cannot sit for long on the chair. In my view, the app should prioritise patient waiting periods in light of their clinical needs and contribute to a more compassionate standard of treatment.” (CRC survivor 2, FG3, male, 58 years).*

Participants expressed the value of a helpline feature that would allow them to connect with the hospital for urgent issues. They also highlighted the importance of an electronic triage function that could be consulted to determine whether a visit to the hospital is necessary or if there are specific steps to be followed, such as “wait and observe” or managing the issue at home. This feature would help eliminate unnecessary visits and provide remote support during the treatment phase after discharge from the hospital.

*“Our time can be saved if we know when to go to the ER and when we do not, but sometimes we do not know who to call and we end up going to the ER because we do not know who to contact, I remember that my family took me to the hospital because of a terrible, smelly wound discharge, when we arrived at the ER they said this is a simple issue that can be managed at day care or at home, but at that time I did not know how to do wound dressings and none of my family members did either, so we ended up going to the ER anyway.” (CRC survivor 3, FG1, female, 66 years).*

*“It’s a good idea to have a helpline so that if there is a significant issue, it may be escalated to the appropriate provider, and a specified time limit should be provided to reach the patients, like six hours or three hours, depending on the scenario.” (CRC survivor 2, FG3, male, 58 years).*

#### 3.2.2 Theme 2: requirements for interactions between HCP-users and peers

A secure interactive patient case manager with three tiers, including a patient interactive educator, symptoms tracker, and interactive communicator functions, would enhance engagement between HCPs, patients, and carers on an interactive platform. This platform would also facilitate connections with peer patients. The interactive educator function would enable the implementation of personalized supportive care plans in a collaborative manner. Additionally, the educator functionality would be designed to cater to individuals with different levels of HL, including the older adult or those with cognitive decline. These tools provide patients with relevant information about their disease and treatment options. Additionally, the educator function employs visualization tools to effectively communicate the risks and benefits associated with different procedures. By facilitating this information exchange, the educator may promote patients’ engagement and active participation in the shared decision-making process while potentially supporting their emotional needs.

*“My dad does not know what carcinoembryonic antigen (CEA) is, only that it’s a blood test to check his disease. We tried to explain, but he did not care.” (IC2, FG1, female, 27 years).*

*“There should be user-enabled content generation based on the patient’s preferences and emotional needs. For instance, the app should be able to generate content for the patient in a way the patient can understand, as well as if it can read aloud the information and explain medical terminology to the patient. You can also allow the patient to enter a set of inputs like stage, type of cancer, user education background, any impairments, and I’m thinking of entering information about my own emotional needs.” (IC3, FG2, male, 36 years).*

*“Some patients prefer not to read, therefore offering alternative formats for information delivery, such as videos or podcasts, is essential. The process by which the content is created is also crucial.” (IC2, FG1, female, 27 years).*

Leveraging e-health solutions to support patients with limited digital skills can be achieved by incorporating their ICs to act as “digital mediators” through a specific interface in the e-health app. A mediator is a newly coined phrase that refers to someone who utilizes their HL abilities to assist others, often somebody with a biomedical background and a medium to high level of education who can explain information to patients and assist them with technicalities (215). Alternatively, digital mediators might be present in the clinic to give patient training and technical help.

*“Because younger caregivers are more digitally skills, they’ll benefit senior patients.” (IC2, FG1, female, 27 years).*

*“I think youngers can use it very well. There’s an educated person in every family who can use it technically. In this generation, a 3-year-old can use a mobile phone and access You Tube by himself. Every home has someone who can use technology and the Internet.” (IC1, FG1, male, 26 years).*

In the treatment phase (surgery and chemotherapy/radiation), the interactive case manager employs an interactive communicator to facilitate real-time communication and problem-solving between patients and HCPs. The communicator function enables multidisciplinary care providers to deliver supportive care interventions, such as patient education and counseling, responsive feedback, and adjustment of supportive care plans based on changing patient needs, through periodic electronic consultations. The role of the communicator can also encompass supporting virtual care, periodic assessments, and providing emotional support to stable survivors. Additionally, the communicator may delegate a specific HCP to contact patients, collect patient feedback, and act as the accountable focal point in communicating with the rest of the care team, advocating for the patient’s needs, and enhancing the team’s response and quality of care. Participants noted that this Patient-HCP communicator could address knowledge gaps caused by care providers’ busy schedules, offer timely assistance, save time, provide assurance, and reduce distress associated with uncertainty.

*“I believe that we need some features to be activated during treatment relating to communicating with the medical teams. Because in real life the doctors share their telephone with us if we need to contact them if something unexpected happens or if we need some help or quick questions, so having features to support communications with providers during treatment and after surgery is essential to have.” (CRC survivor 2, FG2, female, 53 years).*

*“…my chemotherapy was postponed during COVID-19, I’m unsure what to do or how it will affect me, I’m terrified and constantly thinking about it. Will discontinuing chemotherapy due to Covid have an effect on my results? I need to talk to the physicians, but I cannot get in touch with them*.” *(CRC survivor 2, FG2, female, 53 years).*

The interactive feature between the patient and HCP was emphasized by ICs who felt that the effectiveness of the e-health solution hinges on its capacity to offer communication with healthcare teams and consultation with physicians, particularly outside of office hours should anything happen to the patient and they need immediate counsel.

*“Interacting with the doctor is crucial for the patient and his family.” (IC2, FG1, female, 27 years).*

*“These apps could support them and offer the right consultations; a question-and-answer app will help if a patient had a specific symptom or pain and advise him with suitable antibiotic and medicine especially if there is no time for clinic visit or the hospital is far in distance.” (IC3, FG2, male, 36 years).*

The communicator function within the digital platform can also enable patients to connect with peer survivors, fostering mutual support and the exchange of experiences. Smart healthcare networks facilitate connections based on clinical characteristics, allowing patients to share challenges, successes, and coping strategies. Additionally, securely moderated interactive communication channels provide a confidential space for patients to seek psychosocial support from HCPs and fellow survivors. Privacy measures are crucial to protect users’ information and ensure a trusted environment. The integration of the communicator function empowers patients, promotes a sense of belonging, and enhances the overall well-being of CRC survivors.

*“If I’m in stage A for example, I need to see how other people with similar conditions were treated and how they got better as they went through therapy. This will help me a lot and help me understand my condition better. It will also make me less worried because I will learn about other cases that are similar to mine. Of course, I think this should be done under the supervision of a doctor, and the cases should be matched based on the patient’s stage, needs, and requirements, especially if the patient is willing to learn more and is open to the idea.” (CRC survivor 1, FG3, male, 67 years).*

*“Doctors provide important info, but they cannot always be there. Chatting with other patients helps, but it’s not everything. So, let us balance the doctor’s advice and our shared experiences for the best outcome.” (CRC survivor 1, FG2, female, 59 years).*

Participants also recommended interactive patient-directed seminars, educational workshops, or symposia facilitated by cancer specialists to enhance the collective knowledge of cancer survivors. These forums would serve as a platform to replace passive resources like YouTube, offering interactive and patient-centered symposia. The goal is to empower cancer survivors, foster a community of learners, and promote mutual learning and support among participants.

*“I used to watch videos on YouTube for complementary or holistic medicine, or natural healing, fasting to kill cancer, there are lot of blog and channels talking about this, I found out also that many peer patients are looking for this kind or alternative medicine to cope with cancer. We want to hear the patient voice in addition to medical opinion, so why not have experts and cancer patients share their perspectives in recorded online discussions with reputable doctors to answer these questions, like having online seminars for educating patients?” (CRC survivor 2, FG2, female, 53 years).*

The third-tier function of the patient interactive case manage is the symptoms tracker, which allows patients to log their symptoms or side effects, report pain score, and other self-reported measures of health. Participants noted the usefulness of such data input and the possibility to generate trends from them that may be relevant to communicate with their care providers.

*“To help us keep track of symptoms and figure out how to deal with them if they show up by giving us instructions, guidance, or professional advice.” (CRC survivor 1, FG3, male, 67 years).*

Participants emphasized the need for the symptoms tracker to allow them to report unpleasant problems to their clinicians anonymously, therefore security and privacy were stressed in terms of symptoms reporting, especially bowel habits, which are considered embarrassing.

“*Faecal incontinence was one of the symptoms my father was having, but he only told me as I am a doctor and I frequently check on him because he was embarrassed to tell us about it. I think that privately sharing feedback on these symptoms using an app would assist to lessen such embarrassment. Information about how bowel habits change and how to manage it is less likely to be available in forums, particularly from a cultural standpoint, because patients may be embarrassed to share such intimate symptoms there.” (IC2, FG1, female, 27 years).*

#### 3.2.3 Theme 3: patient’s lifestyle and wellness

Considering post-surgery alterations to the digestive tract in CRC patients, the dearth of accessible, informed nutritional professionals, and the challenge of finding tailored online dietary plans, nutritional assistance, diet planning and interventions were identified as critical and significant requirements for CRC survivors. Moreover, the necessity to formulate and achieve behavioral changes in dietary habits, exercise, weight control and fitness aspirations was deemed as crucial. Notable interest in Complementary and Alternative Medicine (CAM) and natural products was distinctly evident among CRC survivors.

*“Patients like us who have bowel cancer face special problems because they often have to have multiple surgeries to remove all or part of their intestines. Because of this, I think we need to learn more about nutritional interventions and how to get used to our new digestive systems after surgery, since people with bowel cancer often have more than one operation. “(CRC survivor 1, FG1, female, 62 years).*

*“I started to lose weight and my appetite within the first few weeks of therapy. Because of my colostomy, I could not eat much after surgery and had to lose a lot of weight. After experimenting with potatoes, soups, and chicken, I found that the bag quickly became overstuffed and had an unpleasant smell, making clean-up a challenge.” (CRC survivor 2, FG1, female, 59 years).*

*“I’d like to know, for example, what diet I should follow. Should I do a detox or an intermittent fast? What kinds of natural remedies could I use? How do I find out more? So, I’d like that to be on the app.” (CRC survivor 2, FG1, female, 59 years).*

#### 3.2.4 Theme 4: platform content and user interface requirements

Having high-quality content that crafts all the information users require and that matches the experiences and needs of CRC survivors and ICs was considered as an integral feature for the design and development of a holistic multiple-component digital platform, as it can positively influence their experiences. The following criteria for the platform were raised by participants: the platform should be user-friendly, patient-centered, trajectory-based, and goal driven, with an embedded support from the medical team as well as frequently asked questions (FAQs).

Caregivers suggested developing a database of frequently asked questions, categorized by disease stage and validated by experts, to maintain credibility and accommodate each patient’s unique needs throughout their care journey. This feature could enhance the overall user experience. Additionally, the inclusion and recommendation of videos and digital patient materials should be tailored to the user’s specific characteristics and preferences.

*“When a patient asks a question, it will be helpful for others if the question and answer are written down; for example, I asked a question and a doctor answered me. The question and answer will be added to the first stage list where other patients’ questions are listed and they can all view this list. And questions from those in stage three are in another bank of questions, and so on…” (IC3, FG2, male, 36 years).*

*“There is a first stage of the condition in which questions must be validated if they are asked for the first time in order to prevent them from being asked again, Even the suggestions or videos should be tailored to the illness stages, with the first stage having its own questions and the second stage having its own.” (IC1, FG2, female, 57 years).*

Furthermore, the user interface should be intuitive, with language that does not induce stress or concern about the prognosis for patients. The content should be tailored to the psychological needs of cancer patients, avoiding any provocation of anxiety, especially if the patient is experiencing a relapse or worsening symptoms.

*“For example, if the material is distressing for the patient, such as information about their progress or if their sickness is worsening, I feel you should contact their carers...” (IC4, FG2, female, 41 years).*

*“My dad has a cell phone, so he could use the app if it’s simple; it’s not only for me to use.” Simple, clear language should be used. It’s tailored for patient follow-up, including all aspects in his profile; Mind/body/diet/exercise.” (IC2, FG1, female, 27 years).*

*“The design, the content, and the language must be appealing so that when he uses it, he will feel spiritually and mentally at ease.” (IC3, FG2, male, 36 years).*

*“Because not everyone is a doctor, it should be basic and easy to understand.” (IC1, FG1, male, 26 years).*

*“Classifying the content in a way that emotionally satisfies users or what the patient likes to see or not.” (IC1, FG1, male, 26 years).*

Caregivers expressed concern that patients might not utilize the app to record their symptoms during treatment, due to being unwell physically or emotionally, or due to their age. Thus, they suggested the idea of delegating some features to enable caregivers to input patients’ data and share it with health providers during treatment and post-operative recovery. Some even went on to suggest that they have a defined role within the app.

*“I doubt patients will log their symptoms on the app, especially those who are old or simply do not trust technology, but as a caregiver, I am in favour of tracking the symptoms and getting trends over time, especially if we can share these symptoms with the doctors. My dad was not engaged with his treatment after surgery and during treatment because he was depressed. If patients observe progress and believe that keeping track of their symptoms would be helpful and have a beneficial effect on their outcomes, they are more likely to participate in the programme.” (IC2, FG1, female, 27 years).*

*“As a carer, I need a strategy and resources to assist me support my dad in her daily problems, answer her questions, and support her emotionally. It’s a great responsibility since I had to look up and read and locate information, which was difficult. The doctors provide treatment, but the family has a lot to do for the patient, so I propose adding caring plan and define our roles and responsibilities, what should we do at each stage, what are typical challenges and how to overcome them.... who should we contact at each stage of her treatment....” (IC1, FG1, male, 26 years).*

Caregivers underscored the significance of their involvement during treatment to help navigate, plan, and monitor the patient’s journey. Involving CRC caregivers through a dedicated interface offering resources and support can empower them while enhancing their capacity to offer practical, emotional, and informational aid to the patients they care for.

*“I think it would be helpful for caregivers during the treatment stage if we could navigate the patient’s journey and plan activities ahead of time. It would also be helpful to know what a caregiver should do and how to do it well to help the patient.” (IC3, FG1, female, 38 years).*

Additionally, participants noted that the app’s content and features should reflect the treatment journey and patient goals. Users advocated for the inclusion of user-generated input planning and goal setting to enable them to enter and determine their long- and short-term goals during each treatment phase. Users and their carers and HCPs should be able to work together within the app to create, revise, and update care plans, especially those including supportive care.

*“When a patient is recovering from surgery, they may only want to use certain features, such as connecting with HCPs, getting recovery tips, accessing psychological resources, dietary issues, and viewing treatment plans. If you ask the patient in follow-up what features they need, such as appointment reminders or scan reminders, they may not want to use them all at once.” (CRC survivor 2, FG2, female, 53 years).*

*“Each patient case is different, so I think to be successful we need to sit with the providers and make a plan then translate it into an electronic plan. If the app is like the apps in the play store, static and not responsive to changes in the patients’ needs over time, it will be abandoned.” (IC 2, FG3, male, 35 years).*

*“Once I open the app, I want to see information about the disease and where he is now, like, Okay, you passed the hardest part, you are now in stage X, and you need to sleep…. So, it should keep the patient interested and motivated by keeping track of their daily progress in their own programmes.” (IC1, FG1, male, 26 years).*

#### 3.2.5 Theme 5: caregivers’ support

Caregivers also pinpointed their own support as a crucial need that a comprehensive digital solution can address. Therefore, the inclusion of psycho-oncological education and assistance for caregivers, particularly those caring for ostomates, along with the management of caregiver tasks, surveillance data, and other follow-up activities, were deemed essential.

### 3.3 Managing caregiving tasks

This function enables the coordination the time and efforts of several caregivers, as well as the delegation of certain responsibilities to those caregivers while keep tabs on their progress.

*“I would much appreciate it if you could include a tool that would allow me to connect with my sister and agree on a caring schedule and plan for my father.” (IC 2, FG3, male, 35 years).*

*“My brothers and I take care of our mom [she has disabilities and hearing impairment], but I generally end up being the one responsible for scheduling doctor’s visits and other care activities. This app would be incredibly helpful if it allowed us to share this information and coordinate our efforts.” (IC 3, FG2, male, 36 years).*

### 3.4 Surveillance planning and follow up timeline

This feature offers timetables and timeframes for surveillance duties with dates and times for the following 5 years post-surgery. It also offers a symptom checker and reporting capability to alert the patient and caregiver to worrying symptoms and how to respond appropriately based on algorithms that would be created and integrated into the symptom tracker function.

*“There is no general and ultimate plan for routine patient or family activities over the next five years. If the application has a detailed plan with specified timetables and exams, the family will know what the patient will do. Also, to notify us about symptoms that can be crucial and I must check them since they may signify a lot, like the condition is returning or to check up with the doctor quickly in most circumstances.” (IC 2, FG3, male, 35 years).*

### 3.5 Psycho-oncological education and support

Caregivers have identified psycho-oncological education and assistance as a necessity for both patients and their loved ones. Supporting caregivers on how to communicate cancer-related concerns, especially ostomy-related ones, to patients need to be a primary focus of the psycho-educational services included to improve the caregiving process and increase patients’ sense of agency.

“*We constantly try to cheer him [his dad] up, to support him mentally, and we have to lie sometimes because we need hopes, even if they are false hopes.... over time, I become depleted, and it is extremely trying to have someone in your family go through suffering. So, I agree that we need resources and educational materials for carers.” (IC1, FG1, male, 26 years).*

*“My mom’s morale was low after she had an ostomy, and it’s been hard on my mind to take care of her. It would be smart to include information on how to care for an ostomy patient properly and tips on how to help him emotionally and mentally. It is also crucial to learn how the patient thinks in order to approach them appropriately.” (IC3, FG2, male, 36 years).*

*“Indeed, her doctors involve us, yet over time it mentally exhausts you...” (IC3, FG2, male, 36 years).*

### 3.6 Stage 2: aligning of bottom-up user requirements with top-down multilayer framework

**Figure 3:**
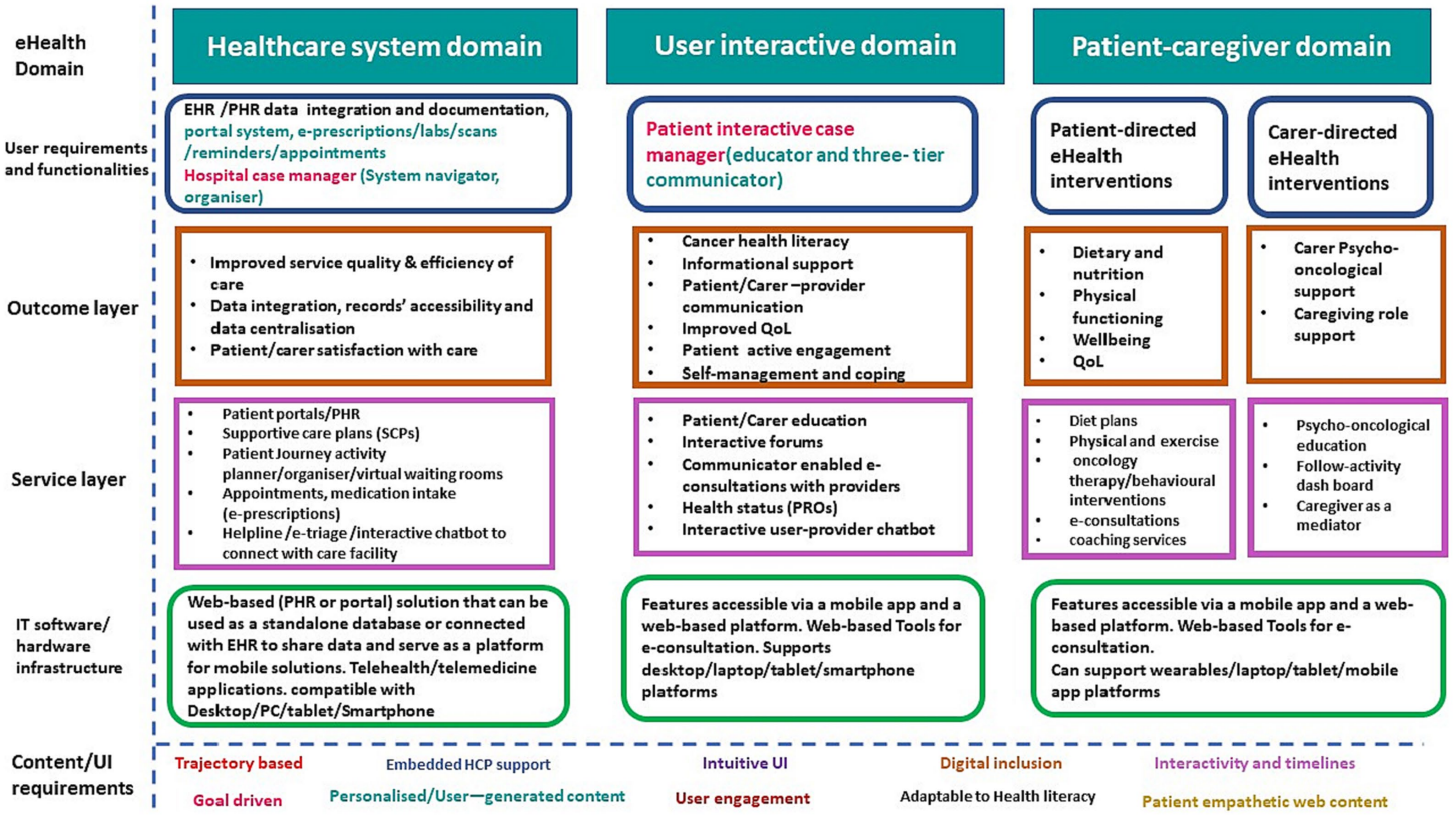
Applying a multilayer framework to design a DMP to support CRC survivors and carers. EHR, Electronic Health Record; PHR, Patient Health Record; SCP, supportive care plan; e-triage, electronic triage; e-prescription, electronic prescription; PC, Personal computer; QoL, Quality of Life; PROs, Patient Reported Outcomes.

In the second phase of the analytical process, the multi-tier framework was meticulously applied. This framework linked the user requirements, which were derived from the initial inductive analysis of the focus group discussions (Figure 1) to the top-down multi-layer framework (Figure 2). The objective of this process was to identify the full scope and domains of the multi-component platform. This alignment of user-centered requirements and functionalities with the expected outcomes, e-health services, and suitable IT software and hardware infrastructure is clearly depicted in Figure 3. This figure elucidates the crucial role of the multi-layer framework in crafting a multi-domain digital solution.

Figure 3 encompasses the application of the multi-tier framework, defining the domains and proposed functionalities of a digital multi-component platform (DMP) designed to support CRC survivors and ICs. The DMP is structured around three primary domains that synthesize bottom-up and top-down user requirements.

The first domain focuses on the healthcare system. Aiming to enhance patient interaction with the healthcare system, improves service quality and efficiency, and streamlines the patient journey and data integration, thus enhancing patients’ satisfaction with the care received. This can be achieved through the integration and documentation of EHR/patient health record (PHR) data, fostering records accessibility and data centralization. The domain comprises a portal system that facilitates e-prescriptions, lab work, scans, reminders, and appointments. It also features hospital case management functionality, including a system navigator and organizer, designed to streamline the patient’s/carer’s journey and follow-up activities during their healthcare facility visits, with the goal of enhancing patient and carer satisfaction. The IT infrastructure underpinning this domain is a web-based (PHR or portal) solution, which can operate as a standalone database or interface with EHR to share data and serve as a mobile solutions platform. It is compatible with desktop/PC/tablet/smartphone platforms and supports telehealth/telemedicine applications. The second domain, known as the user-interactive domain, aligns with the educational and communication needs of patients and ICs. It is facilitated by a patient-interactive three-tier communicator. This communicator includes a patient-HCP communicator to facilitate communications with healthcare providers, and a patient-to-peer communicator to support interactive forums and patient peer groups. The tertiary level of the communicator function facilitates holistic education and health promotion endeavors by employing interactive techniques and leveraging networking opportunities. This is achieved through an array of digital tools, including online discussion forums, symposia, and seminars that aim to foster general education, training, and health promotion. Simultaneously, this platform also ensures the provision of a confidential environment conducive to psychological support, enabling periodic assessments, and extending comprehensive care. This integrated approach strikes a balance between knowledge dissemination and empathetic support, vital for any successful health promotion strategy. The anticipated outcomes of this domain are achieved via a suite of e-health services such as patient/carer education, interactive forums, provider-enabled e-consultations, health status updates PROs, and an interactive user-provider chatbot. The IT infrastructure supporting this domain includes features accessible via a mobile app and a web-based platform and supports desktop/laptop/tablet/smartphone platforms.

The third domain is the patient-caregiver domain, encompassing patient-directed e-health interventions aimed at improving the dietary habits, physical functioning, wellbeing, and QoL of CRC survivors. These outcomes can be achieved through various e-health services such as diet plans, physical exercise regimes, oncology therapy/behavioral interventions, e-consultations, and coaching services. This domain also incorporates carer-directed e-health interventions with the objective of providing psycho-oncological support and enhancing the caregiving role. These outcomes can be fulfilled by a suite of e-health services such as psycho-oncological education, a follow-activity dashboard, and caregiver-as-mediator services. The IT infrastructure supporting this domain is accessible via a mobile app and a web-based platform and can interface with wearable devices and desktop/laptop/tablet/mobile app platforms.

Figure 3 also illustrates the content/UI requirements emphasizing that the DMP should be trajectory based, embed HCP support, has intuitive UI, supports digital inclusion and is adaptable to HL, is interactive and timely, is goal driven, uses personalized/user-generated content, ensures user engagement, and has patient empathetic content. The following features are described below.

#### 3.6.1 Embedded HCP support

The DMP should provide integrated support from healthcare professionals. This could include direct communication tools, decision-making aids, or consultation scheduling functions.

#### 3.6.2 Intuitive user interface

The platform should be user-friendly, with a clear, intuitive design that makes it easy for users to navigate and access information.

#### 3.6.3 Digital inclusion

The DMP should be accessible and usable for users with varying digital skills, including those with limited digital proficiency.

#### 3.6.4 Adaptable to HL

The content should be understandable to users with different HL levels. It may include simplified explanations, visual aids, or options to access more detailed information which can be achieved by digital mediator schema in the DMP.

#### 3.6.5 Interactivity and timeliness

The platform should allow real-time interactions and provide timely responses or feedback to keep users engaged and informed.

#### 3.6.6 Goal-driven

The DMP should support users in setting, tracking, and achieving health-related goals, providing motivation and a sense of progress.

#### 3.6.7 Personalized/user-generated content

The platform should enable users to personalize their experience, contribute their own content, or share their experiences, creating a more engaging and meaningful experience.

#### 3.6.8 User engagement

The platform should incorporate features that encourage regular use and engagement, such as interactive elements, notifications, or gamified aspects.

#### 3.6.9 Patient empathetic content

The DMP should present content that is sensitive and responsive to the emotional and psychological needs of the patients, ensuring that it supports their well-being throughout their cancer journey.

## 4 Discussion

This study employed qualitative methods and a stepwise multi-tier framework to identify the design elements and functionalities of a comprehensive digital platform that supports CRC survivors and their caregivers. By utilizing qualitative methods, the study generated novel findings that contribute to the field by determining user-centered, bottom-up requirements for a collaborative approach in designing a digital platform. These findings further inform the design specifications and functionalities of a holistic, multi-component e-health solution aimed at supporting CRC survivors and their caregivers throughout their care journey.

Furthermore, this research employed a modified multi-tier framework to establish a systematic alignment between user-centered needs, current e-health outcomes, e-health services/interventions, and IT software and hardware infrastructure, ensuring the fulfillment of top-down design criteria. The collaborative design strategy for the DMP employed in this study actively engaged both CRC survivors and caregivers in the design process, to ensure the provision of inclusive support for both groups. Notably, the study emphasized the role of caregivers as digital mediators, underscoring the importance of digital inclusion even for individuals with limited digital skills. This approach aimed to provide equitable access to e-health services for cancer survivors and address varying levels of cancer HL.

CRC survivors showed particular interest in components offering individually relevant information, such as access to EMR, self-management tools, and nutritional and lifestyle advice. On the other hand, ICs were most enthusiastic about caregiving activity management and informational support. They proposed ideas for personalized content delivery, digital inclusion, and content customization aimed at enhancing patients’ HL through the provision of patient-friendly resources like videos, animations, or podcasts.

### 4.1 Digital multicomponent platform features

This study provides valuable insights into the preferences and requirements of CRC survivors and their caregivers when it comes to adopting digital health solutions for streamlining healthcare processes. The findings align with existing literature, indicating a broader trend among patients toward embracing digital health solutions (72). The participants expressed a strong desire for a consolidated health data platform, allowing easy access to medical records and e-prescription refills, to address the fragmented nature of the healthcare system. This aligns with previous research emphasizing the importance of enhancing healthcare efficiency through digital solutions (73, 74). The concept of an integrated digital case manager was highlighted as a means to improve patient outcomes by promoting patient-centered care and empowering users (75, 76). The study also revealed the significance of personalized apps that cater to the specific clinical needs of patients, as well as the inclusion of features like a helpline and electronic triage, which have been associated with better care management and patient satisfaction (60, 61, 62, 77). PHRs or patient portals, integrated with EHR, can support the care needs of CRC survivors. These tools enhance patients’ access to their medical data, laboratory results, scans, and a range of medical services such as e-consultations. PHRs empower patients by centralizing their medical data and enhancing their understanding of their care (78). Moreover, access to personal medical records can improve patient interactions with healthcare professionals, mitigating fragmented cancer care delivery by enhancing communication via portal systems. Haggstrom and Carr’s (62) explored the perceptions of CRC survivors, carers, and health care providers toward a tailored PHR. Their study advocated the necessity of provider buy-in for PHR adoption and emphasized patient and carer involvement. Valued features of the PHR included self-tracking, self-management, and encrypted communication. Although concerns arose about patient access to raw data, PHRs were deemed useful for storing critical information. Patients and carers appreciated the PHR diary for introspection and emotional support. Clinicians viewed the PHR as an informational resource, while patients and carers found it relationally beneficial. Despite the benefits, there are areas that need further exploration for their successful integration into digital supportive care models (78). Furthermore, the study shed light on the requirements for interactions between HCPs and patients/peers on a digital health platform. The participants emphasized the importance of an interactive educator function that supports personalized care plans and imparts disease and treatment information. This finding is consistent with the emphasis on patient-centered care and patient education in the literature (72). The delivery of informational and educational needs in a personalized, non-distressing manner, adaptable to various HL levels, was deemed necessary to enhance the uptake and reap the benefits. HL is an individual, global, contextual, and multifaceted challenge (45, 79). Considering the literacy levels of cancer patients and carers is crucial when designing effective educational programs (44, 45). This is especially pertinent for cancer patients above the age of 65, who constitute a significant proportion of those diagnosed (54). Personalized content based on patient characteristics can be enabled through AI-powered systems (77). Further, the inclusion of a symptoms tracker in the digital platform was identified as crucial for real-time monitoring and self-reported data collection, aligning with the growing recognition of the potential of self-reported data in improving patient outcomes (63).

The study also highlighted the significance of an interactive communicator function, enabling prompt communication between patients and HCPs/peers. This aligns with the value attributed to social support and community engagement in patient care (80). The platform should be relationally beneficial, interactive, and permit networking between patients and carers and between peers and cater to their psychosocial needs and offer coping mechanisms, and practical support. The existing healthcare system often fails to provide sufficient psychosocial support due to increasing numbers of survivors, limited carers, and lack of requisite expertise (64). Caregivers also require social support owing to the demands of their role (45). The study also stressed the importance of lifestyle and wellness issues like nutritional support, dietary planning, and CAM. CRC survivors identified these components as essential, emphasizing their usefulness in digital health solutions. This aligns with the broader literature on the importance of addressing these aspects in cancer survivorship (12, 65, 66, 81).

The findings from this study on platform content and user interface requirements align with the existing literature. Participants emphasized the importance of high-quality content that is user-friendly, patient-centered, and tailored to the needs of CRC survivors and their caregivers. This aligns with research highlighting the significance of personalized digital health solutions and UCD (57, 72, 82). The inclusion of a FAQs database categorized by disease stage reflects the need for credible information and individualized support throughout the care journey (67, 83). The study underscores the necessity of an intuitive, non-stress inducing user interface, especially considering cancer patients’ psychological needs. This aligns with earlier research advocating for Conversational User Interfaces (CUIs) like chatbots and voice assistants, which are gaining ubiquity and evolving for more complex interactions. They exhibit potential benefits for individuals with cognitive decline, the older adults, and the disabled in navigating web and integrated technologies. However, their success hinges on the accessible design. CUIs could enhance accessibility, as demonstrated by interfaces beneficial to cancer survivors (84). Additionally, participants stressed the involvement of caregivers and the need for features that allow them to input patients’ data and support them during treatment, demonstrating the role of caregivers in patient care and the value of their engagement (61). These findings emphasize the need for patient-centered digital platforms that provide tailored content, involve caregivers, and support the holistic needs of CRC survivors and their families.

A DMP can be leveraged to deliver scientifically validated e-health interventions to enhance oncology therapy management, including nutritional (malnutrition or weight gain), psycho-oncology, physical activity, and symptom monitoring. One of the significant challenges is the discrepancy between the speed of software development and the slow pace of clinical trials. While clinical trials are essential, they were designed for static items, like a pill. However, technologies like an e-health platform are dynamic and evolve even during a trial. This requires the incorporation of innovative research concepts without compromising the reliability and accuracy of clinical studies. Distinguishing between evidence-based apps and those lacking evidence is a critical challenge in e-health today. Low perceived clinical effectiveness among physicians and lack of implementation guidelines may threaten the achievement of e-health quality standards, as well as e-health scalability and sustainability (84).

## 5 Conclusion

This study used qualitative methodologies and a multi-tier framework to identify the design features and functions of a digital platform for CRC survivors and their carers. The study’s results highlight the significance of UCD, personalized content, and the engagement of carers in the platform, thereby contributing novel insights. The participants strongly advocated for a consolidated health data platform that is integrated with EHRs. This platform should offer convenient access to medical records, customized care plans, and informative resources. The study emphasized the necessity of interactive features that facilitate communication among patients, HCPs, and peers. Additionally, it stressed the importance of incorporating features that address lifestyle and wellness issues.

## 6 Limitations

Limitations of this study include the application of the theoretical framework to CRC survivors and their informal caregivers in Jordan, an upper MIC Eastern country, which may limit generalizability to other cultural contexts. The design framework requires further validation. Best-worst research or conjoint analysis can be employed to assess preferred features, activities, and recommendations in larger samples of users. Due to time and funding constraints, as well as the impact of the COVID-19 pandemic in terms of access and social restrictions, the research was only able to complete the preliminary stages of the design framework. Future work should include completing the technical design process, conducting usability and feasibility studies, and developing user-centered training materials focused on HL. Proof-of-concept pilot testing is also suggested to evaluate the intervention uptake, retention, and efficacy. Additionally, cross-cultural validation across diverse settings should be conducted. This would facilitate a more comprehensive understanding of how an app if designed performs and is perceived by CRC survivors and caregivers in diverse cultural contexts.

## Data availability statement

The original contributions presented in the study are included in the article/Supplementary material, further inquiries can be directed to the corresponding author.

## Ethics statement

The study was approved by Kingston University’s ethical principles for scientific research (approval num-ber/1416) and Jordan University Hospital’s (JUH) Internal Review Board (IRB), protocol ID (10/2019/8990). The studies were conducted in accordance with the local legislation and institutional requirements. The participants provided their written informed consent to participate in this study.

## Author contributions

SM: Conceptualization, Data curation, Formal analysis, Investigation, Methodology, Software, Writing – original draft, Project administration, Resources, Supervision, Validation, Visualization. RK: Methodology, Resources, Supervision, Validation, Visualization, Writing – review & editing, Conceptualization, Formal analysis.
