# Supplementary material for: Multilayer framework for digital multicomponent platform design for colorectal survivors and carers: a qualitative study
Source: Front Public Health. 2023 Dec 5;11:1272344. doi: 10.3389/fpubh.2023.1272344 (PMC10728820; doi:10.3389/fpubh.2023.1272344)
Supplement: Supplementary file 1 [file Table_1.DOCX]

**Supplementary 1: Participant Information Sheet (PIS) for CRC survivors/ Informal carers’ focus groups**

Study: Determination of User Requirements for a Holistic Digital Platform for Collaborative Patient-Centred Care Models in Oncology

Date: ……/……/ 2019

We are inviting you to participate in a research study. Before deciding, it is crucial for you to understand why the research is being conducted and what it involves. Please take your time to read the following information sheet carefully.

After reading this sheet, if you decide to participate, you will be asked to sign an informed consent form. However, choosing not to participate won't put you at any disadvantage. We appreciate your time considering our project. Should you have any questions or uncertainties after reading this information, we are available to answer them. You should only sign the consent form once your queries have been resolved and you feel comfortable volunteering.

Study Purpose

The primary aim of this study is to conduct a focus group with cancer survivors to gain a deeper understanding of their acceptability and specific requirements for a holistic digital platform designed to support cancer survivors in Jordan.

Why Have I Been Chosen?

As a carer or a cancer survivor, you are a potential participant in this study. Your experience makes you the ideal candidate to help us understand user requirements and guide the development of a holistic digital platform. This platform will support numerous cancer patients and their family members, with an aim to enhance their care and improve their quality of life.

Participation Voluntary

Participation in this research study is entirely voluntary. After reading this information sheet, if you decide to participate, we will ask you to sign a consent form. You can withdraw from this study at any point without disadvantage and without providing a reason.

Study Process

If you decide to participate, you will be asked to join a group discussion with other cancer survivors at a time and date convenient for you. The discussion will focus on gathering your insights and preferences regarding the requirements and potential features of a comprehensive digital platform designed to support cancer survivors in the future.

Benefits

Your participation will be a valuable contribution to our research. Your experiences as a cancer survivor will provide insights for developing a user-centred digital platform catering to the specific needs of Jordanian cancer patients. Identifying these requirements is crucial for the successful development of this platform, which we believe will benefit many cancer patients in Jordan in the future.

Disadvantages and Risks

There are no risks associated with participating in this study. However, we understand that your time is valuable, and we greatly appreciate your contribution.

Post-Study

After the study concludes, you are under no obligation to volunteer again. You can contact the researchers using the contact details provided at the end of this information sheet if you wish to discuss the findings.

Confidentiality

All information collected during the study will be kept strictly confidential and stored securely. Only the main researchers, Samar Melhem and Prof. Reem Kayyali, will have access to this dataset. Any personal information collected will be immediately destroyed, in accordance with the University's research policy.

Organising and Funding

This study is part of a research project, conducted within the School of Life Sciences, Pharmacy, and Chemistry at Kingston University. None of the investigators will financially benefit from this study.

Results

The study's findings will be available at the Faculty of Science Learning Resources Centre (library) at Kingston University. They may also be presented at national and international conferences and published in scientific journals. Your identity will remain confidential as the results will be aggregated for the entire group.

Study Review

The study has been reviewed and approved by the Jordan University Hospital Institution Review Board (IRB) and the Kingston University Faculty of Science Research Ethics Committee. The project is being supervised by Prof. Reem Kayyali.

Contact Information

For further information, you may reach out to:

Samar Melhem

Email: K1831594@kingston.ac.uk

Tel: 00447732720203

Tel: 00962785554464

Project supervisor: Prof. Reem Kayyali

Faculty of Science, Engineering and Computing, Kingston University London

Penrhyn Road, Kingston upon Thames, Surrey KT1 2EE

Email: R.Kayyali@kingston.ac.uk

Tel: +44 (0)20 8417 2561

If you have concerns about any issue raised by your participation in this study, please contact Prof. Reem Kayyali using the provided contact details.

We appreciate you considering participation in this study.
