# Supplementary material for: Multilayer framework for digital multicomponent platform design for colorectal survivors and carers: a qualitative study
Source: Front Public Health. 2023 Dec 5;11:1272344. doi: 10.3389/fpubh.2023.1272344 (PMC10728820; doi:10.3389/fpubh.2023.1272344)
Supplement: Supplementary file 2 [file Table_2.DOCX]

**Supplementary 2 Focus group Topic Guide**

**Aim: To determine the potential features of a mobile application in light of the preferences, perspectives, and experiences of cancer survivors and caregivers.**

1. Experience with Mobile Applications

- Can you tell me about your experience of using mobile applications?

- What types of apps do you use for personal and professional use?

- Have you used health/fitness, medication reminders, or nutrition apps?

2. Role and Importance of Mobile Apps in Cancer Care

- In your opinion, what is the role and importance of mobile apps in supporting cancer patients and their informal caregivers?

- What are the benefits or advantages of such a digital solution as a supportive tool for you as a caregiver throughout the patient journey and treatment plan?

3. Desired Features and Information in the App

- What would you like this app to offer?

- What information would you like the app to provide as a caregiver? (Prompts: information about short and long-term side effects, how to manage between cycles, nutritional plans, effect of cancer on relationships, insurance, appointments, follow-up schedules such as mammograms, ultrasounds, colposcopies, etc.)

- What other features would you desire? (Prompts: communication tool with Healthcare Providers (HCP), side effect alerts, medication reminders, online consultations, communication with HCPs, etc.)

- Would you use it? What makes an app an attractive option to support your journey as a caregiver/cancer survivor?

- At what stage of the journey with the disease would an app be most useful to you and to your patient? (For example, during active treatment or after follow up)

4. Follow-Up Support

- Focusing specifically on follow-up, how can the app improve some aspects of the follow-up? (Prompts: appointments, live tracking of the patient’s journey in the clinic, medication refill, waiting to get your medication ready, medication shortage, communication with HCPs about such issues, etc.)

5. Emotional and Social Support

- With regards to emotional support, how can the application provide emotional-social support to cancer patients?

- To what extent do people like to share their experiences via written and visual content (videos) or simply group meetings with peers? What are your thoughts on this? (Prompts: why, explain, other opinions, etc.)

- What are your recommendations for the content and information needs?

- What are the social and individual barriers that may facilitate or hinder using apps for social support?

6. Potential Disadvantages

- What about the disadvantages or pitfalls of a mobile app for cancer supportive care?

- Will you use it all the time or at certain times? Can you explain why you would continue using it?

- What is the perceived value of different features?

- Do you think that all features will be useful during all phases of the disease?

- Do you think that the mobile app can replace face-to-face consultations or enhance communications?

7. Additional Insights

- Is there anything else you would like to share with us on this topic?

**Thank you for your contribution!**
