# Supplementary material for: Multilayer framework for digital multicomponent platform design for colorectal survivors and carers: a qualitative study
Source: Front Public Health. 2023 Dec 5;11:1272344. doi: 10.3389/fpubh.2023.1272344 (PMC10728820; doi:10.3389/fpubh.2023.1272344)
Supplement: Supplementary file 3 [file Table_3.docx]

**Supplementary 3: Ayyoubzadeh et al. Top-down multilayer approach for e-health development for CRC survivors**

**
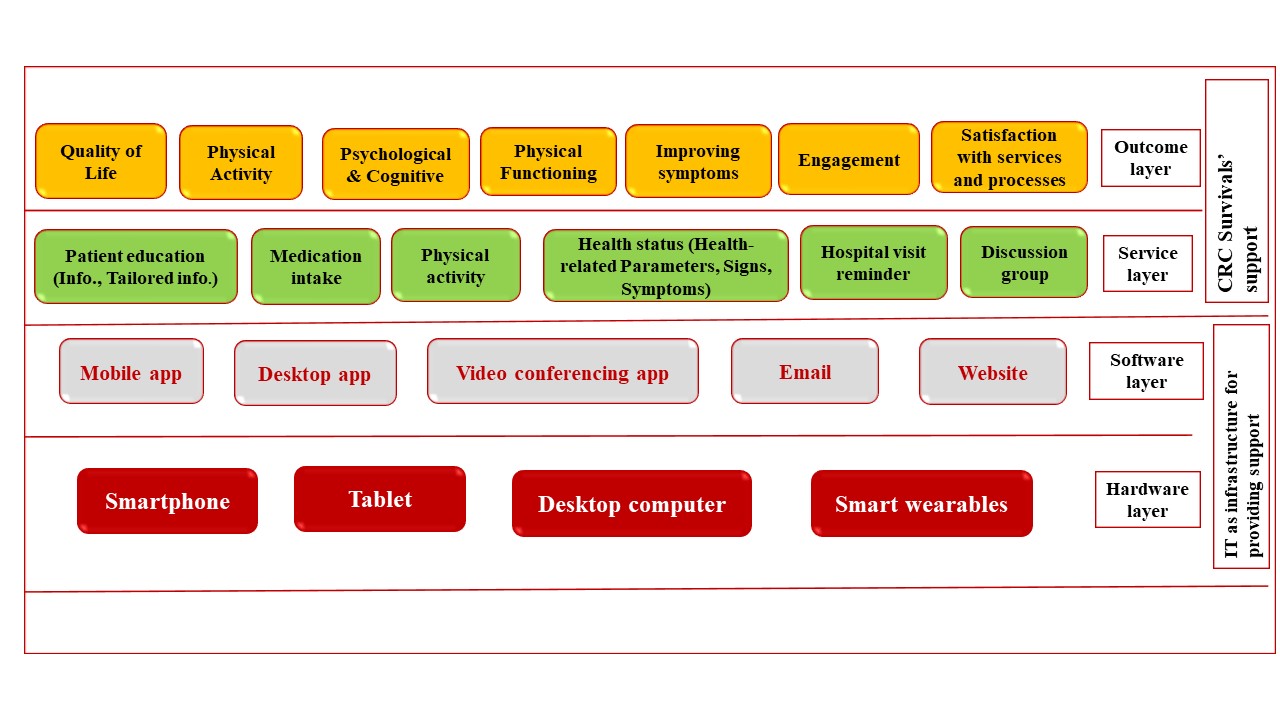
**

**Figure 1_S3: Top-down multilayer approach for e-health development for CRC survivors (60)**

The four-layer structure of Ayyoubzadeh *et al*. (60) summarised in figure 1_S3 consists of the following:

a) The top layer (outcome layer) includes successful results from prior CRC survivor studies. Based on the literature, outcomes of e-health interventions were significantly improved in several areas, including QoL, mental and cognitive health, physical activity and functioning, symptoms, motivation, and engagement.

b) The second layer (service layer) comprises solutions tailored to patients' specific requirements. In this layer, the patient is educated about the disease, other patients' experiences, exercise, controlling relapse, and side effects. It also includes medication intake reminders, physical activity tracking (goal setting and activity recording), health status monitoring (notifications), discussion groups, and consultations with clinical professionals. Physical activity-related services and professional consulting were commonly reported topics, along with providing information and e-PROs and symptom reporting.

c) The software layer represents the system's software architecture that supports CRC survivors' top layer services. It includes mobile apps, desktop apps, websites, video-conferencing apps, email and Web technologies.

d) The hardware layer is the lowest layer, comprising components that enable software programmes to execute. It includes sensors (activity trackers, thermometers, weight scales for cancer survivors) and terminal devices (smartphones, tablets, PCs).
