# Supplementary material for: Multilayer framework for digital multicomponent platform design for colorectal survivors and carers: a qualitative study
Source: Front Public Health. 2023 Dec 5;11:1272344. doi: 10.3389/fpubh.2023.1272344 (PMC10728820; doi:10.3389/fpubh.2023.1272344)
Supplement: Supplementary file 4 [file Table_4.docx]

**Supplementary 4: Systematic flow of bottom-up /top-down approach for the design of e-health tool to support CRC survivors and their ICs**
