# Supplementary material for: Multilayer framework for digital multicomponent platform design for colorectal survivors and carers: a qualitative study
Source: Front Public Health. 2023 Dec 5;11:1272344. doi: 10.3389/fpubh.2023.1272344 (PMC10728820; doi:10.3389/fpubh.2023.1272344)
Supplement: Supplementary file 5 [file Table_5.DOCX]

**Supplementary 5: Consolidated Criteria for Reporting Qualitative Research (COREQ) 32-item checklist CRC cancer survivors and ICs focus groups**

Developed from:

Tong A, Sainsbury Craig J. Consolidated criteria for reporting qualitative research (COREQ): a 32-item checklist for interviews and focus groups. *International Journal for Quality in Health Care.*2007, Volume 19, Number 6: pp.349-357

| **COREQ Criteria** | **Criteria fulfilment in the current research** |
| --- | --- |
| **Domain 1: Research team and reflexivity** | |
| **Personal Characteristics** | |
| 1. Interviewer/ Facilitator | It was noted in the methodology section that the first author (SJM) performed the focus groups |
| 2. Credentials | In the paper, the research team's qualifications and affiliations were listed. |
| 3. Occupation | The research team's occupations were given in the methods section. |
| 4. Gender | The methods section noted that the analysis team comprised of three female academics. |
| 5. Experience and training | The analysis was undertaken by a research team with rich experience performing qualitative research in healthcare settings, as stated in the methods section. |
| **Relationship with participants** | |
| 6. Relationship established | This was not stated in the study. Before the study, none of the research team had relationships with any subjects. |
| 7. Participant knowledge of the interviewer | SJM phoned all eligible participants to clarify the research objectives and answer any questions, and because the interviewer and CRC survivors did not know each other, additionally a brief explanation of the research was given during face-to-face interviews. Caregivers were informed of the study's objectives prior to the Skype focus groups. Researchers emailed or WhatsApped participants a research information sheet. This was in methodology section. |
| 8. Interviewer characteristics | Qualification, occupation, gender, and the absence of a pre-existing link between the interviewer and the interviewees are all provided about the interviewer. The methods section addressed this point. |
| **Domain 2: Study Design** | |
| **Theoretical Framework** | |
| 9. Methodological orientation and theory | Descriptive phenomenology was the main theoretical orientation. Data was analysed, however, using inductive/deductive thematic analysis. This was noted in the methods section. |
| **Participant selection** | |
| 10. Sampling | A convenient sampling strategy of ambulatory CRC cancer survivors and ICs was used, with participants recruited from a large tertiary hospital (JUH) utilising an indirect recruitment strategy by their specialists, as detailed in the methods section. |
| 11. Method of approach | Clinicians approached participants in person or by phone. This was stated in methodology. |
| 12. Sample size | This study included 10 CRC survivors and 10 ICs who took part in focus groups. As stated in the methodology section, sample size was chosen by data saturation 3 online focus groups (comprising 10 survivors) and 3 focus groups comprising 10 ICS were held, and talks were halted when no new fresh ideas for topics were created, indicating thematic saturation. |
| 13. non-participation | All eligible participants who were approached and agreed to participate in accordance with the procedures described in the methods section were enrolled. Thus, the paper did not discuss non-participation. |
| **Setting** | |
| 14. Setting of data collection | focus groups with CRC survivors and ICs were held through Skype. This was mentioned in methodology. |
| 15. Presence of non-participants | Nobody other than the participants in the focus groups was present. |
| 16. Description of sample | Table 1 shows the sample characteristics of CRC survivors while table 2 shows ICs characteristics, mentioned in the results section. |
| **Data Collection** | |
| 17. Interview guide | The topic guide was provided in supplementary 3. |
| 18. Repeat interviews | There were no follow-up interviews, as noted in the methods section. |
| 19. Audio/ visual recording | As stated in the methods, all online focus groups conducted *via* Skype were audio-recorded. |
| 20. Field notes | No handwritten notes were taken. |
| 21. Duration | The length of the online focus groups was specified in the methodology section. |
| 22. Data saturation | Saturation of data was used to determine sample size, and this was documented in the methods section. |
| 23. Transcripts returned | For participants to review and remark on, transcripts were not given back. |
| **Domain 3: analysis and findings** | |
| **Data analysis** | |
| 24. Numbers of data coders | The first author performed data coding. However, the two authors separately inspected the original first author’s coding, and disagreements were discussed. All authors thoroughly debated and approved the coding framework. |
| 25. Description of the coding tree | The "Methods" section describes the code structure and framework. |
| 26. Derivation of themes | Themes were derived inductively from data and from prior concepts literature review. Thus, the authors used hybrid inductive/deductive framework methodology. |
| 27. Software | The *NVivo 12* software was used to manage and code the data. |
| 28. Participant checking | This was not carried out. |
| **Reporting** | |
| 29. Quotations presented | In this paper under the results section, participants' quotes were included. |
| 30. Data and findings consistent | To verify interpretations and findings, the authors evaluated and validated the final themes and subthemes. |
| 31. Clarity of major themes | The thematic analysis weighted all themes equally. |
| 32. Clarity of minor themes | Within the thematic analysis, all themes received equal weighting. |
